# The Influence of CO_2_ and Exercise on Hypobaric Hypoxia Induced Pulmonary Edema in Rats

**DOI:** 10.3389/fphys.2018.00130

**Published:** 2018-02-28

**Authors:** Ryan L. Sheppard, Joshua M. Swift, Aaron Hall, Richard T. Mahon

**Affiliations:** ^1^Department of Submarine Medicine and Survival Systems Groton, Naval Submarine Medical Research Laboratory, Groton, CT, United States; ^2^Department of Undersea Medicine, Walter Reed Army Institute of Research and Naval Medical Research Center, Silver Spring, MD, United States

**Keywords:** vascular reactivity, HAPE, chemoreflex, hypercapnia, exercise

## Abstract

**Introduction:** Individuals with a known susceptibility to high altitude pulmonary edema (HAPE) demonstrate a reduced ventilation response and increased pulmonary vasoconstriction when exposed to hypoxia. It is unknown whether reduced sensitivity to hypercapnia is correlated with increased incidence and/or severity of HAPE, and while acute exercise at altitude is known to exacerbate symptoms the effect of exercise training on HAPE susceptibility is unclear.

**Purpose:** To determine if chronic intermittent hypercapnia and exercise increases the incidence of HAPE in rats.

**Methods:** Male Wistar rats were randomized to sedentary (sed-air), CO_2_ (sed-CO_2_,) exercise (ex-air), or exercise + CO_2_ (ex-CO_2_) groups. CO_2_ (3.5%) and treadmill exercise (15 m/min, 10% grade) were conducted on a metabolic treadmill, 1 h/day for 4 weeks. Vascular reactivity to CO_2_ was assessed after the training period by rheoencephalography (REG). Following the training period, animals were exposed to hypobaric hypoxia (HH) equivalent to 25,000 ft for 24 h. Pulmonary injury was assessed by wet/dry weight ratio, lung vascular permeability, bronchoalveolar lavage (BAL), and histology.

**Results:** HH increased lung wet/dry ratio (HH 5.51 ± 0.29 vs. sham 4.80 ± 0.11, *P* < 0.05), lung permeability (556 ± 84 u/L vs. 192 ± 29 u/L, *P* < 0.001), and BAL protein (221 ± 33 μg/ml vs. 114 ± 13 μg/ml, *P* < 0.001), white blood cell (1.16 ± 0.26 vs. 0.66 ± 0.06, *P* < 0.05), and platelet (16.4 ± 2.3, vs. 6.0 ± 0.5, *P* < 0.001) counts in comparison to normobaric normoxia. Vascular reactivity was suppressed by exercise (−53% vs. sham, *P* < 0.05) and exercise+CO_2_ (−71% vs. sham, *P* < 0.05). However, neither exercise nor intermittent hypercapnia altered HH-induced changes in lung wet/dry weight, BAL protein and cellular infiltration, or pulmonary histology.

**Conclusion:** Exercise training attenuates vascular reactivity to CO_2_ in rats but neither exercise training nor chronic intermittent hypercapnia affect HH- induced pulmonary edema.

## Introduction

Rapid ascent to altitudes greater than 2,500 m by non-acclimatized individuals can induce a form of altitude sickness known as high altitude pulmonary edema (HAPE). Clinical symptoms of HAPE include reduced exercise capacity, tachycardia, dyspnea, cough, frothy sputum, chest tightness (Houston, 1960), crackles on exam and the hallmark bilateral patchy infiltrates observable via chest radiography (Vock et al., 1989). Symptoms typically present and worsen within 2 days of significant altitude exposure in the unacclimatized, particularly when physical exertion is involved. Though HAPE is rare at altitudes below 3,000 m (Maggiorini et al., 1990; Gabry et al., 2003), subclinical HAPE has been estimated to occur in up to 75% of individuals at 4,500 m elevation (Cremona et al., 2002)

HAPE progression is driven by pulmonary vasoconstriction that occurs with prolonged exposure to hypoxia. Pulmonary vascular pressures subsequently increase and can stress the delicate alveolar capillary barrier and induce mechanical stress failure. This pulmonary vascular response likely has multiple driving mechanisms, including increased plasma norepinephrine and sympathetic tone (Duplain et al., 1999), elevated endothelin-1 (Ali et al., 2012; Barker et al., 2016), and decreased levels of exhaled nitric oxide (Duplain et al., 2000; Busch et al., 2001) and nitric oxide metabolite concentrations in both the systemic circulation and bronchoalveolar lavage (BAL) fluid (Swenson et al., 2002; Berger et al., 2005; Bailey et al., 2010). Numerous studies have indicated that HAPE susceptible individuals have a reduced hypoxic ventilation response (HVR) accompanied by lower pO_2_ levels and greater hypoxic pulmonary vasoconstriction than non-HAPE sensitives (Hyers et al., 1979; Matsuzawa et al., 1989; Hohenhaus et al., 1995; Bartsch et al., 2002), though a reduced HVR does not appear necessary for HAPE to develop (Selland et al., 1993). The mechanism of reduced HVR is multifactorial, but blunted central and peripheral chemoreceptor sensitivity likely plays a central role (Albert and Swenson, 2014).

Although there is ample evidence of the impact of hypoxia on chemoreceptor reflex, HVR and HAPE, the impact of hypercapnia is less clear. While it has been reported that HAPE-susceptible individuals demonstrate reduced sensitivity to CO_2_ (Mathew et al., 1983), the effect of chronic CO_2_ exposure on HAPE susceptibility has not been investigated. It is also widely acknowledged that acute exercise exacerbates HAPE symptoms however the role of chronic exercise training in HAPE development is unclear. Exercise training increases cardiac output, which may enhance the heterogeneous pulmonary capillary pressures and leakage induced by hypoxia. Indeed, increased pulmonary fluid and BAL solute concentrations have been confirmed in conditioned endurance athletes after hypoxic exercise (Eldridge et al., 2006), but overall the data is very limited. This study examines the effect of chronic intermittent CO_2_ exposure, with and without concomitant exercise training, on the development of hypobaric hypoxia (HH) induced pulmonary edema in a rat model. We hypothesized that chronic CO_2_ exposure would result in an increased incidence and severity of HAPE, and that this effect would be exacerbated by exercise training.

## Materials and methods

### Animals

All experiments were reviewed and approved by the Walter Reed Army Institute of Research/Naval Medical Research Center Institutional Animal Care and Use Committee in compliance with DoDI 3216.01 and SECNAVINST 3900.38C and all applicable Federal regulations governing the protection of animals in research. The facility is AAALAC accredited, and all animals were maintained under the surveillance of licensed veterinarians. Eight to ten week old male Wistar rats (Charles River Laboratories) were pair-housed at the animal care facility and maintained on a 12 h light/dark cycle and provided standard rodent chow (Harlan Teklad 8604) and water *ad libitum*. Animals were quarantined for 1 week (as per institutional policy) before being randomly assigned to one of 5 groups, *N* = 8 per group (Table 1); (1) sham (no HH, no CO_2_, no exercise), (2) sedentary animals exposed to room air only, (3) sedentary animals exposed to a custom 3.5% CO_2_ gas mix, (4) exercised animals exposed to room air, (5) exercised animals exposed to a 3.5% CO_2_ mix. A separate cohort of animals, *N* = 8/group, was utilized for all CO_2_ reactivity experiments.

**Table 1:** Treatment groups.

**Group**	**HH**	**CO_2_**	**Exercise**
(1) Sham	–	–	–
(2) Sed-Air	+	–	–
(3) Sed-CO_2_	+	+	–
(4) Ex-Air	+	–	+
(5) Ex-CO_2_	+	+	+

*HH, hypobaric hypoxia*.

### CO_2_ exposure and exercise training

A 3.5% CO_2_ level was chosen as the target exposure level with consideration of the following; (1) the US Occupational Safety and Health Administration maximum short-term exposure limit for CO_2_ in humans is 3%, (2) 3–4% CO_2_ is noticeable by human subjects and induces moderate respiratory stimulation, (3) one treatment group would be receiving CO_2_ simultaneously with exercise. For the exercise groups, 3–5 short familiarization runs were performed prior to beginning the 1 month training period in order to identify animals with poor treadmill running compliance. Approximately one in eight animals was removed from the study during the familiarization period due to failure to keep up a steady running pace, repeatedly falling back and touching the end plate (set to deliver a very mild electrical shock), or refusing to move off of the end plate altogether, on two or more occasions. Treadmill exercise training was conducted at 15 m/min, 10% grade, for 1 h/day, 5 days/week for 4 weeks on a metabolic modular treadmill (Columbus Instruments, Columbus OH). The treadmill lanes were set up to an air-tight configuration and either room air or a custom mixture (3.5% CO_2_, 20% O_2_, balance N2) was supplied at a rate of 4 L/min. The 4 L/min flow rate was sufficient to maintain CO_2_ and O_2_ levels within each treadmill chamber at 3.5 and 20%, respectively, throughout the daily training period (9600-A1BTP O_2_/CO_2_ Analyzer, Alpha Omega Instruments, Lincoln, RI). In non-exercising groups the animals were placed on an inactive treadmill ventilated with either room air or the CO_2_ mix for 1 h/day.

### Hypobaric hypoxia (HH)

**Figure 1:**
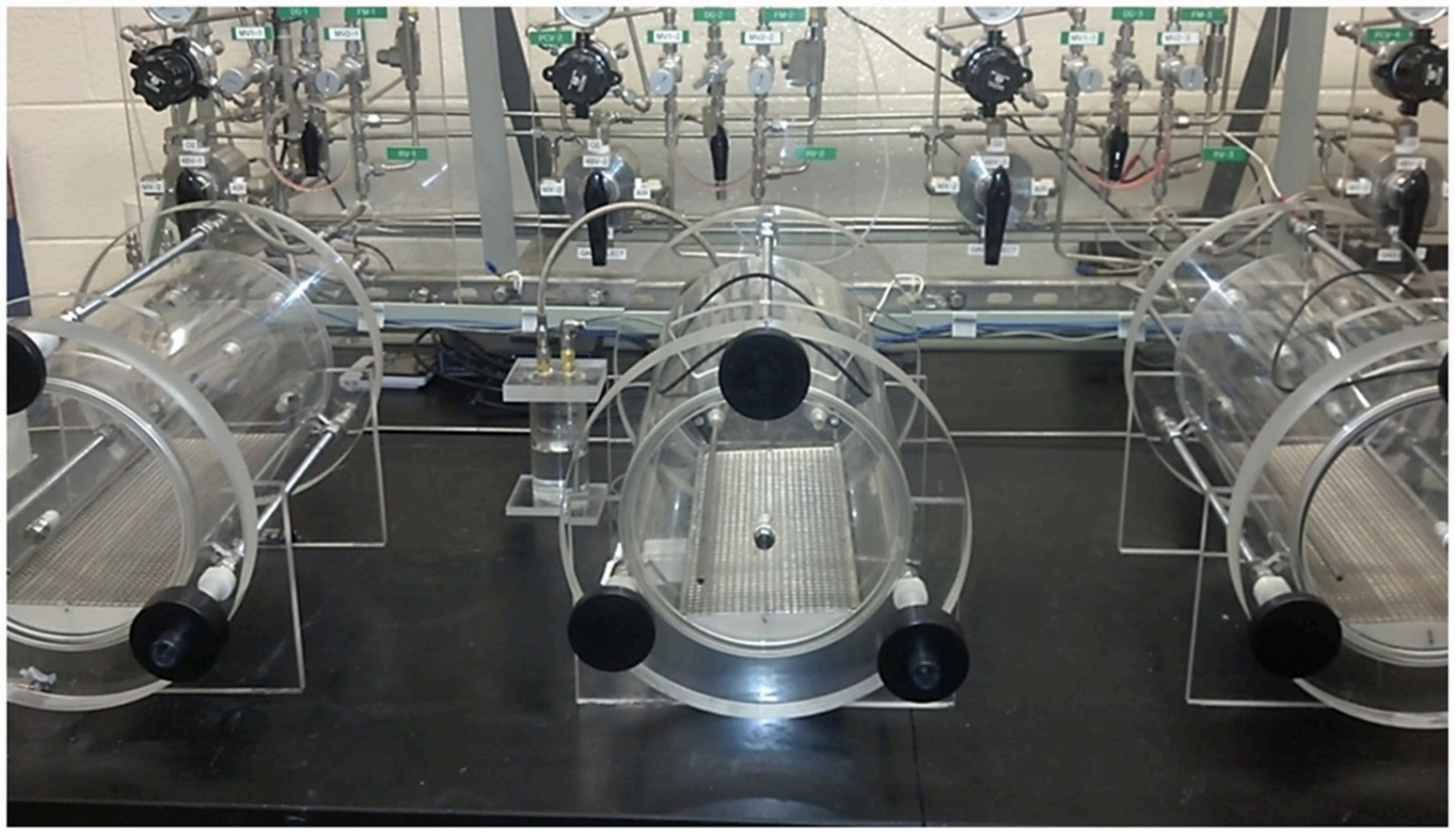
Hypobaric chambers.

Custom-built methylacrylic cylinders measuring 38 cm in length by 20 cm in diameter were constructed in house (Figure 1). Evacuation of the chambers was driven by a GAST 6066 vacuum pump (GAST Manufacturing, Benton Harbor MI) through a side port. Fresh air was supplied at a rate of 2–2.5 L/min from compressed air banks; this flow rate was sufficient to prevent any buildup of CO_2_ (9600-A1BTP O_2_/CO_2_ Analyzer, Alpha Omega Instruments, Lincoln, RI). Chamber temperatures during operation did not differ significantly from the ambient room temperature (23–26°C). Chambers were furnished with rodent chow, water and a hide box for each animal to reduce overall stress. An atmospheric pressure equivalent to 25,000 ft of altitude (282.0 torr, CPA2501 digital air indicator, Mensor, TX) was chosen as the target for HH exposure. We arrived at this value after extensive model development testing revealed that this pressure reliably induced a significant increase in mean lung wet/dry weight ratios, whereas greater atmospheric pressures did not reliably increase wet/dry ratios after 24 h (data not shown). Longer duration exposures were not employed due to extreme dehydration (animals refused fluids during the exposures), and further reduced atmospheric pressures dramatically increased mortality. During all experiments the chambers were de-pressurized/re-pressurized at an equivalent rate of 1,000 ft/min. Intake/outtake valves were adjusted to maintain the targeted altitude equivalent of 25,000 ft ± 500 ft at all times. After 24 h, chambers were re-pressurized (1,000 ft/min) and animals were euthanized by overdose of a sodium pentobarbital solution (150 mg/kg, IP) upon return to sea level. Sham group animals were placed in the hypobaric chambers for 24 h with air flow of 2–2.5 L/min, but the chambers were not subjected to vacuum and remained at ambient room pressures.

### Vascular CO_2_ reactivity

Cerebral blood flow (CBF) following acute exposure to CO_2_ was assessed after completing the 4-week training program via rheoencephalography (REG) as described (Ainslie and Duffin, 2009). Animals were anesthetized via urethane (1,000 mg/kg, IP) and electrodes were prepared and placed as previously described (Bodo et al., 2005). After electrode positioning baseline REG readings were recorded. A 12% CO_2_/20% O_2_ gas mixture was then supplied for 30 s, followed by a 5 min recovery period on room air. The CO_2_ pulse was repeated a second time, and changes in REG amplitude were averaged for each animal.

### Lung tissue preparation and bronchoalveolar lavage (BAL)

Tracheal cannulas were placed and tied in and lungs were removed en bloc within 10 min of returning to ambient room pressure. A small portion of each right lobe was flash frozen in liquid N_2_ for later gene expression analysis, while the majority was weighed, dried at 55°C for 72 h, and weighed again to determine the wet to dry weight ratio. BAL samples were obtained by gently infusing 1 ml of PBS into the left lung a total of 3 times, pooled, and stored on ice. Portions of each left lobe were preserved in a 10% formalin solution, sectioned 5 μm thick at equidistant points using a microtome and tissue block, hematoxylin/eosin stained, and examined by a board certified veterinary pathologist for evidence of fibrin thrombi, edema, endothelial damage, cellular infiltrates, thickening of the alveolar septum, hemorrhage or necrosis. Tissues were assigned quantitative values for each category on a 0–3 scoring system; 0 = no significant lesions, 1 = ≤ 20% of tissue affected, 2 = 20–50% of tissue affected, 3 = ≥ 50% of tissue affected. To ensure accuracy in scoring, 5 individual sections per animal were examined. Lavage fluid was spun at 1,500 g for 5 min, and the resulting pellets were then re-suspended in 1 ml of ice-cold PBS and immediately analyzed for cell type (Advia 120 Hematology System, Siemens Healthcare, Tarrytown, NJ). The supernatant was analyzed for lactate dehydrogenase activity and total protein content (Pierce BCA Protein Assay, Thermo Scientific, Rockford, IL).

### Lung vascular permeability

Lung vascular permeability was examined *in vivo* after exposure to HH or normobaric conditions for 24 h. Sodium fluorescein dye (5 ml/kg, 2% solution; F6377-500G; Sigma-Aldrich, St. Louis, MO) was injected through the tail vein in a separate cohort of rats immediately after normobaric or hypobaric exposure. Thirty minutes after fluorescein injection rats were anesthetized with a ketamine/xylazine cocktail (80 and 10 mg/kg, respectively, IP) and transcardially perfused with PBS to remove the fluorescent tracer prior to harvesting the lungs. Lungs were rinsed with cold saline and homogenized with 2 ml of PBS. After homogenization, 2 ml of 50% TCA solution was added and the samples were vortexed to mix for 30 s to precipitate proteins in the lung samples. The resultant sample solution was cooled at 4°C for 30 min and covered to ensure no light exposure to the sample. Upon completion of cooling the samples were centrifuged for 10 min at 3,300 × g in 4°C. One mL of supernatant from each sample was placed into a 1.5 ml microcentrifuge tube and centrifuge at 16,000 × g at 4°C for 10 min. 200 ul of supernatant from each sample was added to a 96-well microplate along with 12 standards and read via spectrofluorometer according to manufacturer directions. Results are expressed as relative fluorescence units per liter.

### Analysis

All data are expressed as means ± SEM and analyzed using GraphPad Prism (GraphPad Software, La Jolla, CA). Two-way ANOVA (sedentary/exercised X air/CO_2_) was used to analyze for main effect and interactions between factors for each dependent variable. Tukey's *post-hoc* test with multiplicity adjusted *P*-value was used for pairwise comparisons. The sham group was not included in ANOVA as comparisons of sham vs. HH with CO_2_/exercise trained groups were not of interest. Instead, unpaired *t*-tests were used to determine the effects of HH (sham vs. sed-air only) on each dependent variable. Statistical significance was accepted at *P* ≤ 0.05.

## Results

### Rat HH induces pulmonary edema

**Figure 2:**
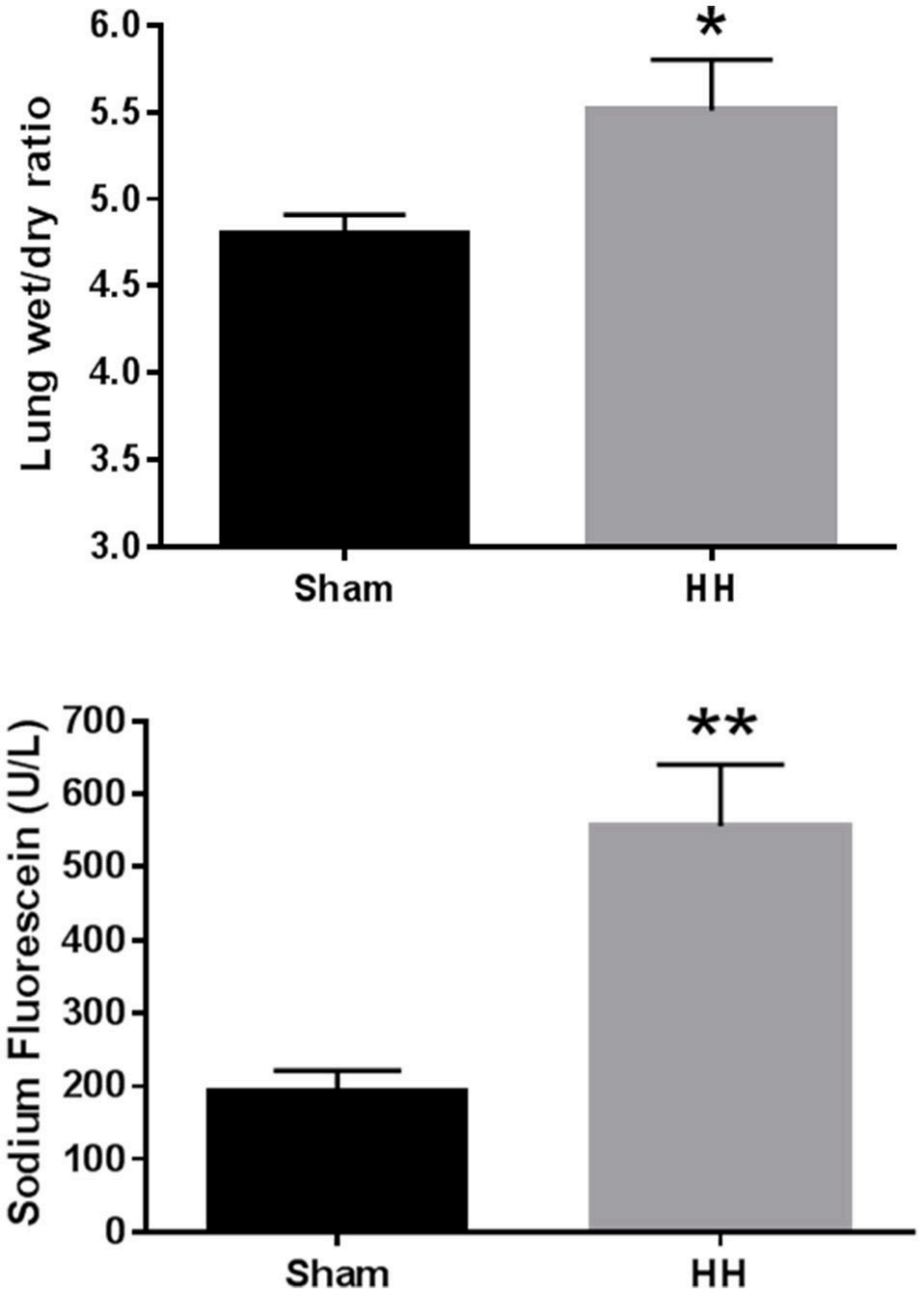
Effects of HH on pulmonary fluid accumulation (**A**, top) and pulmonary vascular permeability (**B**, bottom). Animals were exposed to atmospheric pressure equivalent to 25,000 ft elevation or ambient pressure for 24 h. ^*^P < 0.05, ^**^P < 0.001. *N* = 8 per group.

HH exposed animals exhibited signs of pulmonary injury consistent with HAPE. Lung wet-to-dry weight ratios in sed-air animals increased 15% over sham animals (*P* = 0.014; Figure 2). Pulmonary vascular permeability in the sed-air group, assessed by sodium fluorescein dye leakage, was increased 190% over sham (*P* < 0.001). Total protein content, white blood cell concentration, and platelet concentration in BAL fluid were significantly increased by HH (Table 2), while there was no change in lactate dehydrogenase activity in the BAL fluid, and no red blood cells were detected in either group. Histological examination revealed no evidence of gross pulmonary pathology.

**Table 2:** Hypobaric hypoxia exposure vs. sham control.

	**Sham**	**Sed-air**	**Sed-CO_2_**	**Ex-air**	**Ex-CO_2_**
**MASS**					
Pre	391 ± 8 g	464 ± 17 g	458 ± 15 g	464 ± 17 g	464 ± 17 g
Post	375 ± 7 g	423 ± 16 g	420 ± 14 g	423 ± 16 g	423 ± 16 g
Δ	16	41	37	37	36
% change	(−4.3%)	(−8.9%)^**^^a^	(−8.0%)	(−9.2%)	(−9.2%)
**BAL**					
Protein (μg/ml)	114 ± 14	222 ± 33^**^^a^	171 ± 22	151 ± 14	186 ± 21
LDH (U/L)	261 ± 13	305 ± 31	269 ± 29	274 ± 29	274 ± 26
Platelet (1 × 106^∧^3/uL)	6.0 ± 0.6	16.4 ± 2.3^**^^a^	11.8 ± 1.3	8.8 ± 1.1^*^^b^	11.8 ± 1.2
WBC (1 × 10^∧^3/uL)	0.68 ± 0.07	1.16 ± 0.26^*^^a^	0.79 ± 0.09	0.47 ± 0.07^*^^b^	0.69 ± 0.10
RBC	ND	ND	ND	ND	ND

ND, not detected,

* P ≤ 0.05,

** P ≤ 0.001, in compares on to

a sham,

b *sed-air. Hypobaric hypoxia groups are compared to each other; Sham is compared to sed-air group only*.

### Exercise training attenuates CO_2_-induced increases in CBF

**Figure 3:**
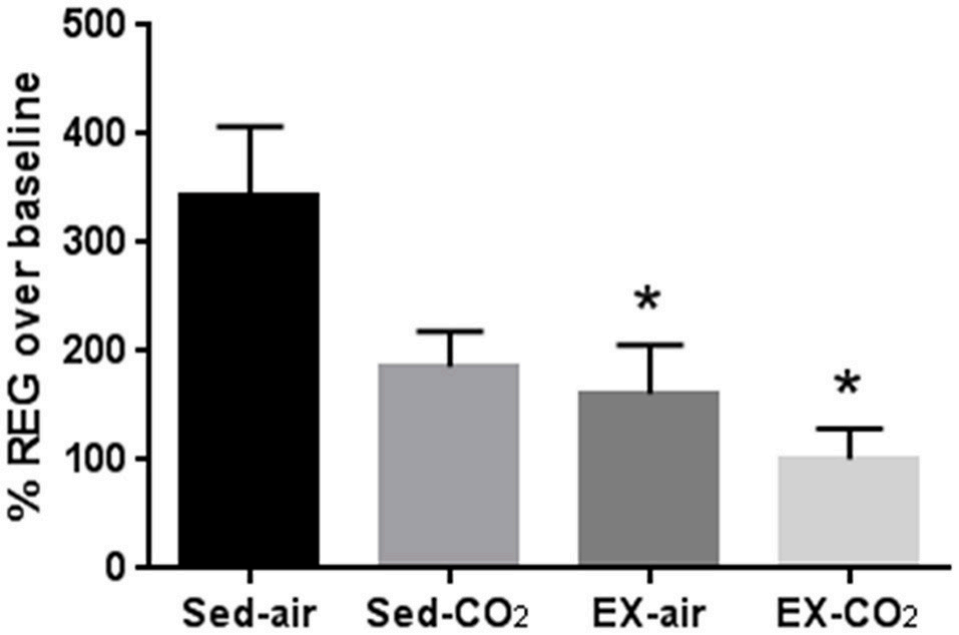
Changes in cerebral blood flow after acute exposure to 12% CO_2_. Rheoencephalogram amplitude after acute CO_2_ exposure was compared to baseline recordings on room air. *Indicates significant difference (*P* < 0.05) from sedentary-air group. Sed-air (*N* = 6), sed-CO_2_ (*N* = 8), ex-air (*N* = 6), ex-CO_2_ (*N* = 7).

Exposure to a 30 s, 12% CO_2_ pulse increased REG amplitude by 343% ± 63.9, 185% ± 33, 161% ± 45, and 100% ± 28, in sed-air, sed-CO_2_, ex-air, and ex-CO_2_ groups, respectively (Figure 3). Daily CO_2_ exposure (sed-CO_2_) resulted in an apparent reduction in the CO_2_ pulse-induced REG increase but this effect did not reach statistical significance (*P* = 0.062), while exercise alone (ex-air) and exercise w/CO_2_ (ex-CO_2_) attenuated the CO_2_-pulse induced REG increase by 53% (*P* = 0.038) and 71% (*P* = 0.003) vs. sed-air, respectively. REG values in the sed-CO_2_, ex-air, and ex-CO_2_ groups were not significantly different from each other and there was no interactive effect. One animal in the ex-air group was removed from the study due to a foot injury that precluded treadmill running, and two animals in the sed-air and one animal from the ex-CO_2_ group died due to complications from anesthesia and/or REG electrode placement.

### CO_2_ and exercise training do not affect the incidence or severity of HH-induced pulmonary edema

**Figure 4:**
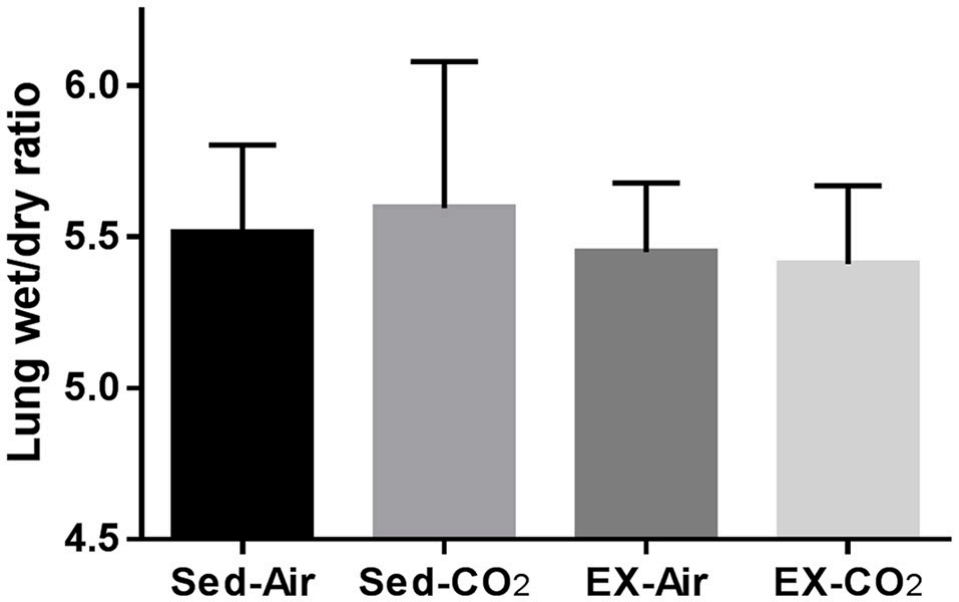
Effects of HH on pulmonary fluid accumulation after 1 month of exercise and CO_2_ exposure. Results are following exposure to atmospheric pressure equivalent to 25,000 ft elevation for 24 h. Sed-air (*N* = 8), sed-CO_2_ (*N* = 8), ex-air (*N* = 6), ex-CO_2_ (N = 8).

**Figure 5:**
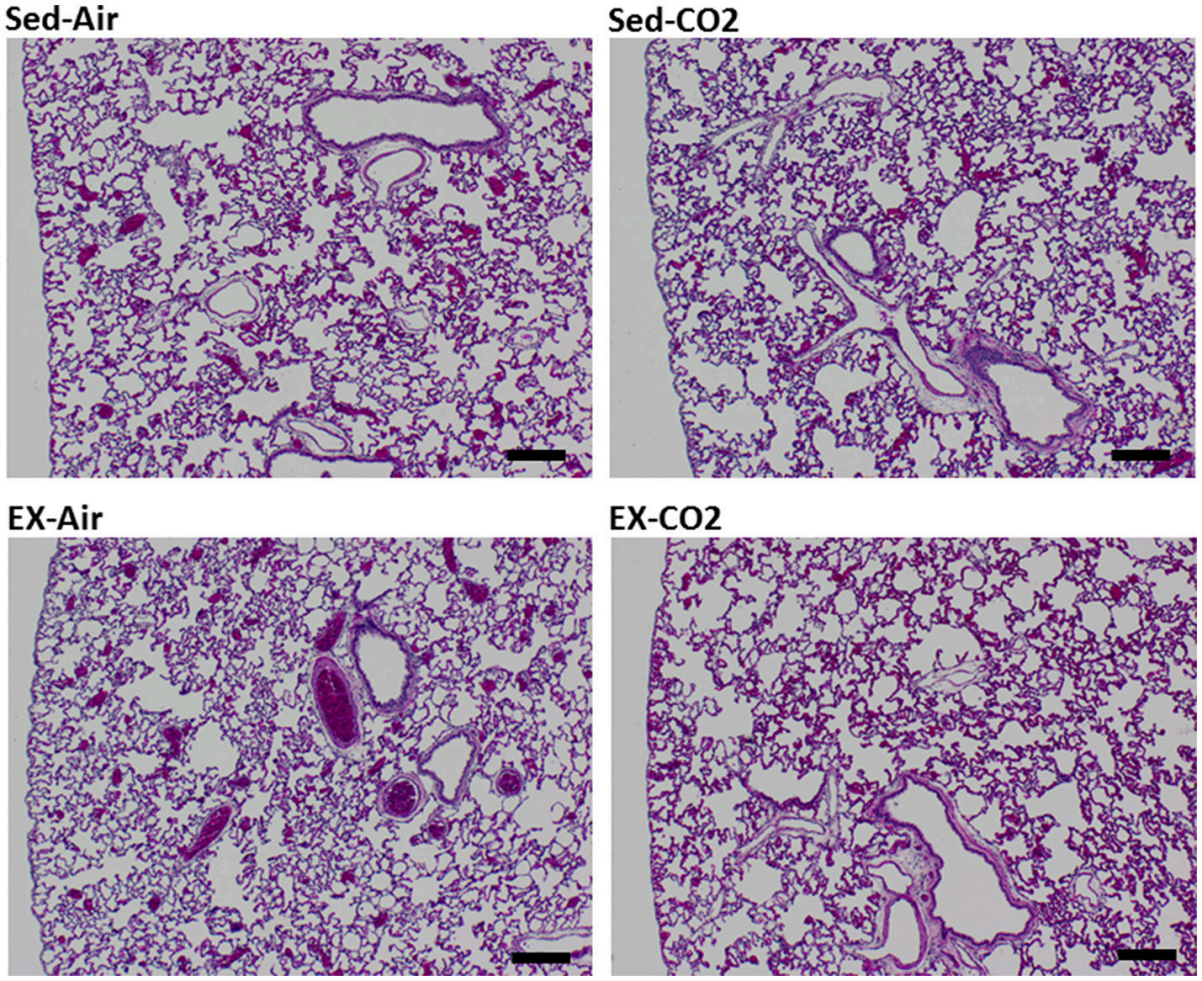
Histology of HH exposed left lung lobe. Representative sections for each treatment group. Tissues were assigned quantitative pathology scores by a veterinary pathologist. Black bar = 200 μm.

To assess the effects of chronic exercise and CO_2_ exposure on the development of HAPE in rodents, we exposed each of the four groups to HH equivalent to 25,000 ft of altitude for 24 h. Though the lung tissues were edematous in comparison to non-altitude exposed tissues, exercise and CO_2_ exposure had no effect on between group wet-to-dry weight ratios (Figure 4). BAL analysis revealed no difference in total protein content or LDH activity between any of the groups however the ex-air group did have decreased platelet and total white blood cell concentrations in the BAL fluid (Table 2). Upon histological examination the tissues from each group were indistinguishable and relatively unremarkable, showing minimal signs of cellular infiltrates, septal thickening, hemorrhage, or necrosis (Figure 5).

## Discussion

The primary aim of this study was to determine how chronic intermittent CO_2_ exposure may affect HAPE susceptibility in rats. HAPE development is largely dependent upon an inappropriately high level of pulmonary vascular constriction concurrent with exposure to hypoxia. Repeated hypoxic exposures appear to alter the chemoreceptor reflex (Berkenbosch et al., 1992; Mahamed and Duffin, 2001), and we hypothesized that chronic intermittent CO_2_ exposure would also affect chemoreceptor sensitivity, exacerbate the pulmonary vasoconstriction effect of hypoxia, and worsen the symptoms and severity of HAPE. We also hypothesized that chronic CO_2_ would reduce HVR, further exacerbating HAPE development. As most individuals acutely exposed to altitude are also performing relatively strenuous exercise (mountaineers, extreme sport athletes, military personnel, etc.) an exercise component was included to assess the interaction of chronic intermittent CO_2_ and cardiovascular conditioning on HAPE susceptibility. The results presented here do not support our hypotheses, but do include some interesting findings.

Our HAPE model successfully induced pulmonary edema where previously published efforts to develop rat models of HAPE have met with mixed success. The methodology and exposure protocols have varied considerably, and while some studies have reported significant changes in wet-to-dry weight ratios after various altitude exposure profiles (Shukla et al., 2011; Lee et al., 2013), others did not demonstrate any differences in wet/dry ratio (Omura et al., 2000; Berg et al., 2004), used other methods to identify and quantify pulmonary edema (Li et al., 2011; Lin et al., 2011), or used very severe exposure protocols to induce pulmonary edema (Bai et al., 2010). Though our model was generally well tolerated the animals were lethargic, did not consume food or water during the exposure, and lost a significant amount of bodyweight (see Table 2). Despite a robust HAPE model however, exercise training and chronic intermittent hypercapnia did not affect HH-induced pulmonary edema. It is possible that simulated altitude of 25,000 ft for 24 h (greater than what most humans are likely to experience) was sufficient to elicit a maximal level of pulmonary vascular stress and that any effects of hypercapnia and exercise were masked or comparatively minor. Lower pressures and/or longer exposure times dramatically increased mortality and morbidity, which lends some support to this hypothesis. A more modest simulated altitude exposure may allow for more differentiation between the treatment groups, but our preliminary tests at higher atmospheric pressures were insufficient to reliably induce an appreciable level of pulmonary edema.

The level of bodyweight loss in HH-exposed animals was quite striking with most animals losing ~9% of total bodyweight in 24 h. Similar bodyweight losses have been reported in mice after exposure to ~14,000 ft of altitude for 3 days, however most of this was due to adipose lipolysis (Hannon and Rogers, 1975) which is unlikely to account for much of the losses here, and previous studies have reported reduced fluid intake in rodents exposed to altitude (Jones et al., 1981). The bodyweight reductions seen here were likely mostly due to loss of total body water, however preloading the animals with subcutaneous fluid (25–30 ml normal saline) and humidifying the supply air to ~60% during HH also had no discernible effect on bodyweight loss or lung wet/dry weight ratios in a separate cohort of animals. Sham animals that were not exposed to HH averaged 4.3% loss of total bodyweight over the same time period, indicating roughly half of the bodyweight reduction can be attributed to non-HH factors. Nevertheless the additional bodyweight reductions seen in HH-exposed animals may still have been sufficient to appreciably reduce plasma and interstitial fluid volume. As HAPE is driven by heterogeneously high pulmonary vascular pressures, such a reduction in total blood volume may have been sufficient to attenuate HH-induced increases in pulmonary vascular pressures. We did not perform direct measurements of pulmonary artery pressure or compare pre-post-total body water levels, so this possibility cannot be ruled out.

We hypothesized that chronic CO_2_ exposure would suppress respiratory drive with HH and the study was designed with the intent to periodically measure respiration throughout the HH exposure period. Despite successful preliminary optimization runs, complications during the collection period made the respiratory data unreliable and it was not feasible to repeat the experiments. This is a significant limitation of the study and without this data we can only speculate, however the central and peripheral chemoreceptors responsible for respiratory regulation are highly sensitive to pCO_2_ /H^+^ (Nattie, 1999) and a stimulus that was sufficient to alter the CBF response to hypercapnia could be expected to also affect respiratory drive.

While direct measurement of pulmonary artery pressure via catheterization or echocardiogram would have been preferable, we used CBF as a general indicator of a systemic vascular response to CO_2_, and not as a surrogate of pulmonary vascular reactivity. Exercise markedly suppressed CO_2_-mediated increases in CBF, indicating that the vasculature was not responding to the increased pCO_2_ levels with the degree of dilation seen in control animals. There are several potential mechanisms for this. An increase in circulating H^+^ resulting from CO_2_ metabolism would normally have a direct effect on peripheral vascular smooth muscle leading to vessel dilation (Kontos et al., 1977), but chronic exercise and CO_2_ exposure may have desensitized the vasculature to elevated H^+^ and disrupted normal cerebrovascular autoregulation. Alternatively, CO_2_ and H^+^ can induce formation of reactive oxygen and reactive nitrogen species and may influence brainstem mediated adaptations to hypercapnia and hypoxia (Dean, 2010). Though both cerebral and pulmonary tissues demonstrate a high degree of blood flow autoregulation, local responses to hypoxia are quite different and it is possible this extends to hypercapnia as well. Further investigations comparing vascular autoregulation in the brain vs. lung would be informative. Our findings may also be of interest in the context of High Altitude Cerebral Edema, which was not studied here.

## Conclusion

Chronic intermittent CO_2_ exposure and exercise training do not affect the incidence of HAPE in rats, but exercise training does attenuate CO_2_ induced increases in CBF.

## Author contributions

RS, AH, and RM were responsible for study design and conceptualization. RS and JS collected and analyzed the data. RS compiled the initial manuscript. RS, JS, AH, and RM revised the manuscript and approved the submitted version.

### Conflict of interest statement

The authors declare that the research was conducted in the absence of any commercial or financial relationships that could be construed as a potential conflict of interest.
